# The Value of Indocyanine Green Image-Guided Surgery in Patients with Primary Liver Tumors and Liver Metastases

**DOI:** 10.3390/life13061290

**Published:** 2023-05-31

**Authors:** Benjamin Weixler, Leonard A. Lobbes, Luis Scheiner, Johannes C. Lauscher, Sebastian M. Staubli, Markus Zuber, Dimitri A. Raptis

**Affiliations:** 1 Department of General and Visceral Surgery, Charité-Universitätsmedizin Berlin, Corporate Member of Freie Universität Berlin and Humboldt-Universität zu Berlin, Hindenburgdamm 30, 12203 Berlin, Germany; 2 Department of Hepato-Pancreatico-Biliary Surgery and Liver Transplantation, Royal Free Hospital, London NW3 2QG, UK; 3 Clarunis University Center for Gastrointestinal and Liver Diseases, St. Clara Hospital and University Hospital Basel, 4058 Basel, Switzerland

**Keywords:** indocyanine green, liver surgery, colorectal liver metastases, liver cancer, image-guided surgery

## Abstract

Introduction: Successful R0 resection is crucial for the survival of patients with primary liver cancer (PLC) or liver metastases. Up to date, surgical resection lacks a sensitive, real-time intraoperative imaging modality to determine R0 resection. Real-time intraoperative visualization with near-infrared light fluorescence (NIRF) using indocyanine green (ICG) may have the potential to meet this demand. This study evaluates the value of ICG visualization in PLC and liver metastases surgery regarding R0 resection rates. Materials and Methods: Patients with PLC or liver metastases were included in this prospective cohort study. ICG 10 mg was administered intravenously 24 h before surgery. Real-time intraoperative NIRF visualization was created with the Spectrum^TM^ fluorescence imaging camera system. First, all liver segments were inspected with the fluorescence imaging system and intraoperative ultrasound for identification of the known tumor, as well as additional lesions, and were compared to preoperative MRI images. PLC, liver metastases, and additional lesions were then resected according to oncological principles. In all resected specimens, the resection margins were analyzed with the fluorescence imaging system for ICG-positive spots immediately after resection. Histology of additional detected lesions, as well as ICG fluorescence compared to histological resection margins, were assessed. Results: Of the 66 included patients, median age was 65.5 years (IQR 58.7–73.9), 27 (40.9%) were female, and 18 (27.3%) were operated on laparoscopically. Additional ICG-positive lesions were detected in 23 (35.4%) patients, of which 9 (29%) were malignant. In patients with no fluorescent signal at the resection margin, R0 rate was 93.9%, R1 rate was 6.1%, and R2 rate was 0% compared to an ICG-positive resection margin with an R0 rate of 64.3%, R1 rate of 21.4%, and R2 rate of 14.3% (*p* = 0.005). One- and two-year overall survival rates were 95.2% and 88.4%, respectively. Conclusion: The presented study provides significant evidence that ICG NIRF guidance helps to identify R0 resection intraoperatively. This offers true potential to verify radical resection and improve patient outcomes. Furthermore, implementation of NIRF-guided imaging in liver tumor surgery allows us to detect a considerable amount of additional malignant lesions.

## 1. Background

Approximately 50% of colorectal cancer patients are diagnosed with liver metastases (CRLM) at some point in their disease history [1]. Survival rates of patients with surgically resectable CRLM are reported to be as high as 50%, and 10-year survival rates currently reach 17% [2,3,4,5,6]. Current European guidelines therefore suggest resection of synchronous and metachronous CRLM if R0 resectability can be achieved [7].

Primary liver cancer (PLC) (hepatocellular carcinoma and cholangiocarcinoma) is rare compared to CRLM [8]. Complete surgical resection of PLC is often the only option to achieve a potential cure or at least prolong survival. As with CRLM, satisfactory disease outcome depends on early diagnosis and R0 surgical resection [9].

Other cancer types, such as neuroendocrine tumors, pancreatic cancer, or uveal melanoma, also metastasize into the liver. In uveal melanoma, up to 50% of affected patients will develop distant metastases, of which 90–95% involve the liver [10,11,12]. If metastatic removal is a therapeutic option for these patients, R0 resection should be sought.

Surgeons must rely on intraoperative ultrasound and palpation of the liver to locate the tumor intraoperatively. Intraoperative ultrasound can assist in localizing the tumor but, like palpation, has no value in the determination of R0 resection. Currently, there exists no intraoperative imaging method that could provide reliable information about resection margins.

Real-time intraoperative visualization with near-infrared light fluorescence (NIRF) using indocyanine green (ICG) is a technique used for different clinical applications, such as visualization of bowel perfusions, testing of liver function, and detection of sentinel lymph nodes [13,14,15,16,17,18]. Indocyanine green is mainly bound to serum albumin after intravenous injection. If exposed to NIR light it emits fluorescence that peaks at 840 nm [19]. Light at a wavelength of 840 nm is almost not absorbed by water or hemoglobin and structures that contain ICG can therefore be visualized up to 5–10 mm through body tissue. After intravenous injection, ICG is exclusively excreted by the liver into the bile with a half-life time of two to three minutes [20]. Interestingly, ICG remains around liver metastases for days to weeks and appears as rim-type fluorescence [20,21,22]. This phenomenon is due to dedifferentiated hepatocytes surrounding the metastasis [23,24]. In PLC, the tumor itself accumulates ICG; therefore, it presents less rim-type fluorescence but more staining as a whole, although this is not a general rule [25,26]. Indocyanine green may therefore facilitate R0 resection, as its tissue penetration depth of up to 10 mm could help to identify (too) close resection margins intraoperatively. For years, the impact of resection margin width on disease-free and overall survival was a matter of debate. However, recent evidence demonstrates that resection margin width is an independent predictor of disease-free and overall survival, and resection margins should be >1 cm when feasible [27].

In this study, we investigate the value of intraoperative ICG visualization with NIRF for the definition of oncological resection margins. We then evaluated its significance in the detection of additional hepatic tumor lesions.

## 2. Materials and Methods

### 2.1. Study Design and Population

In this prospective non-randomized cohort study, resection of CRLM, PLC, and metastatic liver disease from other solid tumor types was performed in open or laparoscopic surgery. Inclusion criteria were age ≥ 18 years, ability to provide written informed consent, diagnosis of colorectal liver metastases, a cholangiocarcinoma or a hepatocellular carcinoma, possibility of surgical resection and an ASA score ≤ 3. Exclusion criteria were benign liver tumors, coexisting malignancy of other etiology, liver dysfunction with a model for the end stage of liver disease (MELD) score > 10, known allergy to ICG, iodine, iodine dyes, or drugs known to interact with ICG (e.g., anticonvulsants, bisulfite, narcotics, methadone, nitrofuratoin), as well as pregnancy and breastfeeding.

### 2.2. Ethical Approval

The study was approved by the local ethical review committee (EA4/157/18) and was in accordance with the Declaration of Helsinki (1975) and its later amendments. Written informed consent was obtained before ICG administration on the day before surgery.

### 2.3. Procedure

**Figure 1:**
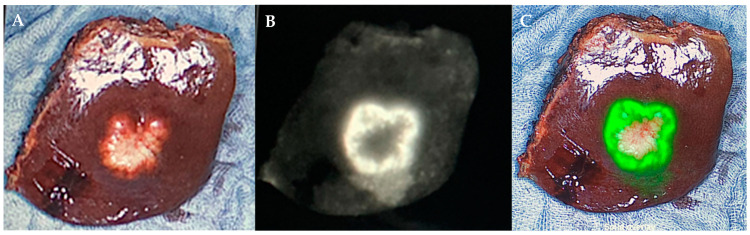
ICG-positive colorectal cancer liver metastasis showing fluorescent rim. (**A**) Color image of colorectal cancer liver metastasis; (**B**) near-infrared light image of colorectal cancer liver metastasis; (**C**) merged image of colorectal cancer liver metastasis.

Dose and time of administration were optimized in a previously published study by van der Vorst et al. [24]. Indocyanine green (VerDye, Diagnostic Green GmbH, Aschheim Germany, 25 mg vials) was dissolved in 5 mL sterile water to yield a 5 mg/mL concentration, and a bolus of 2 mL containing 10 mg ICG was administered 24 h prior to surgery. Real-time intraoperative visualization was undertaken using the Spectrum^TM^ fluorescence imaging platform (Quest Innovations, Middenmeer, The Netherlands). In the first step, all liver segments were inspected with the fluorescence imaging system and with intraoperative ultrasound (Figure 1). Magnetic resonance imaging (MRI) of the abdomen with liver-specific contrast agent was performed as a standard procedure within 4 weeks prior to surgery. If additional superficial lesions were detected, they were intraoperatively resected if practicable and safe regarding the bleeding risk and the remnant liver function. All CRLM, PLC, and liver metastases from other cancer types were resected according to oncological principles. All resected specimens were analyzed back-table in the operation-room with the Spectrum^TM^ fluorescence imaging camera for fluorescence spots at their resection margin immediately after resection, and fluorescent spots were marked with a suture for further pathological analysis (Figure 1). Definition of an ICG-positive margin was the detection of fluorescence at the resection margin. If an ICG-positive signal was detected, this was always considered as an ICG-positive resection margin, regardless of the brightness of the signal. If a positive signal was detected on the resection margin of the resected specimen, no change in operative strategy was made. Distance to tumor tissue for marked fluorescence spots, as well as for fluorescence negative surface, was indicated in mm in the pathological report. Clinical and histopathological data were collected from all patients. R1 resection was defined as microscopic margin involvement.

### 2.4. Follow-Up

A follow-up was performed according to German guidelines, and patients were seen at the university surgical outpatient clinic at the intervals specified for each tumor. Follow-up data were therefore collected from the clinic’s own database.

### 2.5. Statistical Analysis

Statistical analysis was performed using R version 3.3.2 (R Core Team, GNU GPL v2 License) and R Studio version 1.0.44 (RStudio, Inc. GNU Affero General Public License v3, Boston, MA, USA, 2016) with the graphical user interface (GUI) rBiostatistics.com alpha version (rBiostatistics.com, London, UK, 2017) [28].

## 3. Results

### 3.1. Patients Characteristics

A total of 66 patients were included in this study. Patient characteristics are listed in Table 1. Of the 66 patients, 28 had rectal cancer metastases, 20 had colon cancer metastases, 5 had hepatocellular carcinoma (HCC), 5 had uveal melanoma metastases, 4 had cholangiocarcinoma, 2 had neuroendocrine tumors, 1 had metastasis of a parotid carcinoma, and 1 had metastasis of an ovarian carcinoma.

### 3.2. Intraoperative NIRF ICG Visualization

Intraoperative NIRF ICG visualization detected additional ICG-positive lesions compared to preoperative MRI images in 23 (35.4%) of patients. Of these 23 patients, 9 (29%) had malignant tissue in the additionally detected lesions. These account for 13.6% of all patients, in which additional malignant lesions were detected with ICG only. The other 14 (21.2%) patients with additional ICG-positive lesions were false positive. Histology of these additional false positive lesions identified six as bile duct tissue, five as necrosis, five as fibrosis, two as cysts, two as normal liver, one as inflammation, and one as steatosis. In 18/23 patients, one additional lesion was detected, two additional lesions in 5/23 patients, and three additional lesions in 1/23 patients.

On the resected liver specimens, an ICG-positive resection margin was detected in 14 (21.9%) patients. The ICG-positive margin was R0 in nine (64.3%), R1 in three (21.4%), and R2 in two (14.3%) (*p* = 0.005) patients (Table 2). If no ICG signal was detected on the resected liver specimen, the resection margin was histologically R0 in 46 (93.9%) and R1 in 3 (6.1%) patients (*p* = 0.005).

### 3.3. Survival

Median postoperative follow-up was 16 months (IQR 8–24 months). One- and two-year overall survival rates were 95.2% (95% CI 85.7–98.4%) and 88.4% (95% CI 75.6–94.7%), respectively.

**Figure 2:**
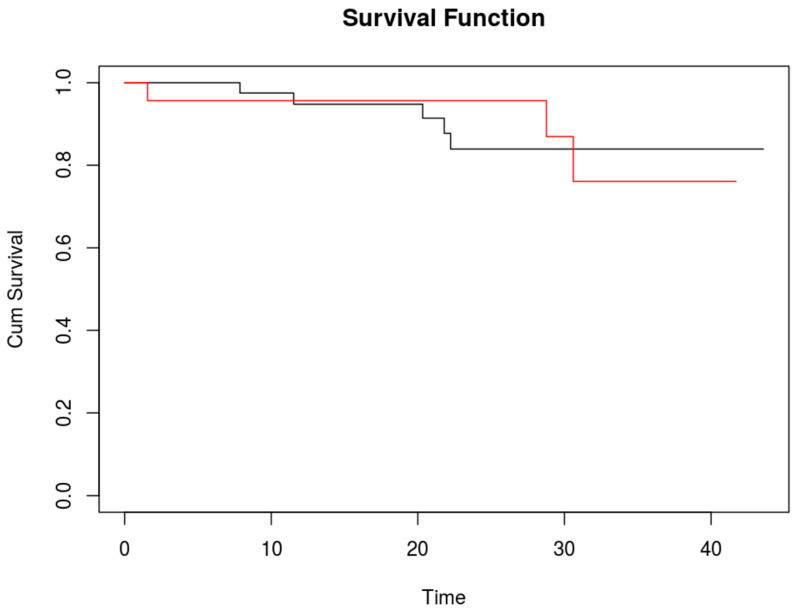
Kaplan–Meier curve for overall survival; red line shows patients with additional malignant ICG-positive lesions, black line shows patients without additional malignant lesions. 95%CI: 0.2353 4.141.

No difference in survival was detected between patients with additional positive ICG lesions and those without (Figure 2). The two-year overall survival rate for patients without additional ICG-positive lesions was 83.9% (95%CI 65.1–93.1%), and for those with additional ICG-positive lesions it was 87% (95%CI 54.3–96.9%).

## 4. Discussion

Achieving R0 resection is of central importance in the treatment of liver metastases and PLC. This study demonstrates that NIRF imaging with ICG can help to accurately identify R0 resection intraoperatively. Recent data illustrated that the R1 rate in conventional open and laparoscopic liver surgery is approximately 14% [29]. In contrast, our data show an R1 of 6.1% when the resection margin is negative for ICG-positive spots. On the other hand, if the resection margin was ICG positive, only about two-thirds of these patients had a histologically tumor-free resection margin. As ICG has a tissue penetration depth of up to 10 mm, not every ICG-positive spot on the resection surface of the liver specimen will necessarily indicate tumor infiltration. However, it informs the surgeon that the tumor will be within 0 to 10 mm from the resection margin and would therefore indicate that it required closer investigation and greater caution when dissecting. In these circumstances, fresh frozen sections from the area of interest in the remnant liver could be performed to obtain better certainty about the resection distance. This approach was not part of this study but was tested recently in a study by Achterberg et al., were persistent fluorescence in the wound of the residual liver was always positive for tumor tissue [30]. Most interestingly, Achterberg reported that if no fluorescent signal was present on the margin of the resected specimen, then a tumor-negative resection could be predicted in the operating room in almost all cases.

Near-infrared light image-guided surgery can direct the surgeon directly to the target area on the liver if the tumor is located on the liver surface or is at least 10 mm in depth. Even for tumors located deeper in the liver ICG has advantages, as its appearance will inform the surgeon of a transection line relatively close to the tumor. It therefore has considerable potential to guide the surgeon in achieving an R0 resection.

One third of the patients in this study showed additional fluorescent spots on the liver surface that were not detected by an MRI preoperatively. A significant number of these ICG-positive lesions were malignant. Almost identical findings were published by a Dutch group investigating the long-term follow-up after ICG-guided CRLM resection, where additional metastases were found in 9 of 67 patients [31]. Handgraaf et al. investigated if the additional resection of these CRLMs has an impact on survival. They compared this group of patients with a group operated on without NIRF-guided surgery but failed to detect differences in survival. The data presented here also failed to show an advantage for survival with regard to the resection of additionally detected liver metastases. As already mentioned by Handgraaf and colleagues, a relatively small sample size could have impacted these results. In order to clarify this important question, larger studies must be carried out in the future.

In one fifth of our patients, ICG visualization did reveal false-positive lesions. In the early literature of ICG fluorescence imaging, the false-positive rate was as high as 40%, double what we experienced in our study [26,32]. However, recent studies in this field seem not to have any false-positive lesions, mainly due to study-specific selection criteria, or they may fail to report them. In their work on real-time identification of liver tumors with ICG, Ishizawa et al. hypothesized that false-positive findings could be reduced if ICG is not administered a day before surgery but with a longer time interval, especially in patients with decreased liver function [26]. In the study by Tummers et al., the authors hypothesized that scar tissue or regenerative tissue of the liver retains ICG by accumulation inside immature hepatocytes [33]. We found positive lesions in fibrotic and cirrhothic lesions, as well as in inflamed and steatotic tissue, therefore confirming the assumptions of these studies. In another study by Ishizawa investigating NIRF imaging for CRLM and hepatocellular carcinoma, a rather high dose of ICG was used (0.5 mg/kg) 2–14 days prior to surgery [26]. Such high doses lead to a passive ICG accumulation in the tumor caused by the enhanced permeability and retention (EPR) effect. ICG is bound to serum proteins and behaves as a macromolecule, accumulating in tumor tissue due to increased vascular permeability and reduced drainage [33]. This effect could possibly allow us to differentiate ICG-positive tumor tissue from healthy tissue. However, we administered a much lower dose at only 10 mg per patient and could easily identify PLC. The administration of lower ICG doses 24 h prior to surgery, as undertaken in this trial, still can detect PLC, most probably due to the EPR effect. One must be aware that the type of fluorescence (i.e., rim-type or staining as a whole [EPR]) cannot accurately distinguish between the etiology of the tumor. As demonstrated in the work by Kokudo, PLC can show rim-type fluorescence according to the grade of tumor differentiation [21]. It is important to note for surgical practice that if ICG stains the tumor as a whole and not as a rim-type fluorescence, surgical resection must be approached with even greater caution since the distance to the tumor will be a bit smaller.

The implementation of NIR “image guided surgery” in operative practice has the potential to enhance radical resection of liver metastases and PLC and therefore to improve patient outcomes. Most studies in this field, however, focus on the detection of superficial CRLM and do not report on the value of ICG for the detection of positive resection margins [34]. Studies on the value of ICG for resection of tumor lesions located deeper in the liver are still missing. Moreover, there are almost no studies focusing on the value of ICG in PLC resection. However, the promising results of the existing studies together with the results presented here give a hint of the great potential of NIRF imaging with ICG for the detection of positive resection margins in liver metastasis and PLC surgery.

## 5. Conclusions

This study provides significant evidence that ICG guidance facilitates R0 resection and helps to identify positive resection margins intraoperatively. This offers true potential to verify radical resection and improve patient outcomes. Our results furthermore demonstrate that NIR-guided imaging in liver tumor surgery detects a considerable amount of additional malignant lesions. A randomized, controlled trial comparing intraoperative ICG visualization is needed to assess its importance in resection margins as well as in disease-free and overall survival.

## Figures and Tables

**Table 1:** Patient characteristics.

Patient Characteristics	*n*
**Age**	65.5 years (IQR 58.7–73.9)
**Sex**	
Female	27 (40.9%)
Male	39 (59.1%)
**Type of surgery**	
Open	48 (72.7%)
Laparoscopic	18 (27.3%)
**Type of operation**	
Extended right	11
Extended left	6
Right hemihepatectomy	7
Left hemihepatectomy	2
Multiple segmentectomy	7
Segmentectomy	13
Wedge	20
**Tumor type of metastases**	
Rectal cancer	28
Colon cancer	20
Hepatocellular carcinoma	5
Uveal melanoma	5
Cholangiocarcinoma	4
Neuroendocrine tumor	2
Parotid carcinoma	1
Ovarian carcinoma	1
**Patients with additional ICG-positive lesions**	23
**Additional ICG-positive lesions:**	
Malignant no	14 (71%)
Malignant yes	9 (29%)
**Child–Pugh Score**	
No cirrhosis	64
B	2
**ASA Score**	
1	3
2	27
3	36
**BMI**	23.7 (IQR 21.2–26.5)
**Length of hospital stay**	9 days (IQR 6–17 days)
**INR preoperative**	1.01 (IQR 0.96–1.06)
**INR 24 h postoperative**	1.31 (IQR 1.14–1.41)
**INR at discharge**	1.08 (IQR 1.02–1.16)
**Postoperative complications (Clavien–Dindo)**	
0	27
1	7
2	8
3a	6
3b	12
4a	2
5	4

Abbreviations: ICG, indocyanine green; ASA, American Society of Anesthesiologists; BMI, body mass index.

**Table 2:** ICG-positive margins in the resected liver specimen.

ICG-Positive Spots at Resection Margin on Liver Specimen	Type of Resection		
	R0	R1	R2
No	93.9%	6.1%	0%
Yes	64.3%	21.4%	14.3%

Perarson’s Chi-squared test *p* = 0.005. Abbreviations: ICG, indocyanine green.

## Data Availability

Not applicable.
